# Predictors of Linkage to Care for Newly Diagnosed HIV-Positive Adults

**DOI:** 10.5811/westjem.2015.4.25345

**Published:** 2015-06-22

**Authors:** Erika Aaron, Tyler Alvare, Ed J. Gracely, Ralph Riviello, Amy Althoff

**Affiliations:** * Drexel University College of Medicine, Division of Infectious Diseases and HIV Medicine, Philadelphia, Pennsylvania; † George Washington University School of Medicine and Health Sciences, Washington, DC; ‡ Drexel University School of Public Health, Epidemiology and Biostatistics, Philadelphia, Pennsylvania; § Drexel University College of Medicine, Department of Emergency Medicine, Philadelphia, Pennsylvania

## Abstract

**Introduction:**

Linkage to care following a human immunodeficiency virus (HIV) diagnosis is critical. In the U.S. only 69% of patients are successfully linked to care, which results in delayed receipt of antiretroviral therapy leading to immune system dysfunction and risk of transmission to others.

**Methods:**

We evaluated predictors of failure to link to care at a large urban healthcare center in Philadelphia in order to identify potential intervention targets. We conducted a cohort study between May 2007 and November 2011 at hospital-affiliated outpatient clinics, emergency departments (EDs), and inpatient units.

**Results:**

Of 87 patients with a new HIV diagnosis, 63 (72%) were linked to care: 23 (96%) from the outpatient setting and 40 (63%) from the hospital setting (ED or inpatient) (p<0.01). Those who were tested in the hospital-based settings were more likely to be black (p=0.01), homeless (p=0.03), and use alcohol or drugs (p=0.03) than those tested in the outpatient clinics. Patients tested in the ED or inpatient units had a 10.9 fold (p=0.03) higher odds of failure to link compared to those diagnosed in an outpatient clinic. When testing site was controlled, unemployment (OR 12.2;p<0.01) and substance use (OR 6.4;p<0.01) were associated with failure to link.

**Conclusion:**

Our findings demonstrate the comparative success of linkage to care in outpatient medical clinics versus hospital-based settings. This study both reinforces the importance of routine opt-out HIV testing in outpatient practices, and demonstrates the need to better understand barriers to linkage.

## INTRODUCTION

Major advances in human immunodeficiency virus (HIV) treatments have reduced morbidity, improved survival, and resulted in millions of years of life saved.^1^–^2^ Substantial efforts to prevent the spread of HIV and morbidity of those infected have relied on increasingly aggressive prevention strategies, including widespread testing. We now know that HIV-positive individuals receiving antiretroviral therapy (ART) are 96% less likely to infect their partners than individuals receiving primary care alone.^3^ To benefit optimally from these advances, HIV-infected persons must know their HIV status, be successfully linked to outpatient care, adhere to ART, and remain engaged in care. Unfortunately only about half of those with HIV are engaged in care, and only 20% of the U.S. population with HIV is virally suppressed on ART.^4^ Failure to link to high quality HIV care after diagnosis prevents the U.S. from achieving successful markers of HIV treatment and the possibility of eliminating HIV from the population.^5^ Delayed linkage to care is associated with delayed receipt of ART leading to immune damage and higher rates of failure to achieve virologic suppression.^6^ Failure to achieve virologic suppression not only affects individual health outcomes, but has critical public health implications by contributing to a high HIV community viral load and allowing for secondary HIV transmission.^7^ Additionally, it is common that newly diagnosed persons with HIV are diagnosed late in the disease process as measured by a low CD4 count (<200cells/μL) and/or an acquired immune deficiency syndrome (AIDS)-defining condition.^8^ The U.S. National HIV/AIDS Strategy has set as goals to decrease the estimated 56,000 new cases of HIV annually by increasing HIV serostatus awareness to 90% and increasing linkage to care to 85% within three months of diagnosis by 2015.^9^ To accomplish these goals, strategies to diagnose and ensure linkage of persons into HIV care are critical.

Drexel University College of Medicine (DUCOM) and Hahnemann University Hospital, a large urban healthcare center in Philadelphia, instituted non-targeted opt-out HIV testing programs in the emergency department (ED) in 2006 and subsequently in hospital inpatient units and outpatient primary care clinics in order to increase HIV awareness and capture HIV-positive patients. The aim of this study was to determine variables that are associated with failure to link to care. We examined socioeconomic and biomedical predictors of linkage to care for newly diagnosed HIV-positive adults. Model results are presented for prediction of failure to link to care, rather than successful linkage, because it is the failure to link that is critical to identify.

## METHODS

We conducted a retrospective cohort study using medical records for persons newly diagnosed with HIV between May 2007 and November 2011 in both outpatient primary care clinics and in hospital settings (ED and inpatient units). All testing sites used antibody testing technology: an initial rapid HIV test followed by a confirmatory Western Blot test if positive. A positive Western Blot was presumed to equal HIV-positive in our study. All analysis was limited to persons who had reported a previous negative test or had reported never having been tested for HIV. Newly diagnosed patients were referred to the co-located DUCOM HIV clinic using one of two procedures: 1) an HIV case manager met the patient at the testing site, a follow-up appointment at the co-located HIV clinic was scheduled within one week of diagnosis and/or hospital discharge, and the HIV case manager met the patient at the first HIV clinic appointment; 2) alternatively, patients diagnosed by clinicians in the ED after clinical hours, when HIV clinic staff were unavailable, were given an appointment date and time in the HIV clinic within one week of diagnosis. An HIV outreach worker attempted to contact the patient by phone to confirm this appointment. Of note, all HIV-positive patients admitted to the hospital were seen by the inpatient HIV consult service, which is staffed by infectious disease specialists who work at the same outpatient HIV clinic. In addition, outreach workers and case management staff attempted to contact all patients who missed their initial or follow-up appointments.

Patients who were tested in all three locations were from the same urban geographic area and lived within a three-mile radius of the HIV clinic. Linkage to care was assessed at the co-located HIV clinic: successful linkage was defined by at least one visit with an HIV medical provider within six months of receiving an HIV-positive test result. Procedures followed were in accordance with the ethical standards of the Institutional Review Board at DUCOM.

### Definition of Variables

All data were ascertained from patient medical records. Variables are defined as follows:

Homelessness: Patients who were living in a shelter or on the street at the time of diagnosis.
Substance Use: Patients with documented drug or alcohol use by positive urine screen, or documentation of substance use in patient medical record within the one year prior to diagnosis.
Mental Illness: Diagnosis was defined as those with documentation of a chronic mental illness. (The most common were schizophrenia, bipolar disorder, and major depression.) Episodic conditions such as a single depressive episode were not included.
CDC AIDS Category: This variable was dichotomously defined using the Centers for Disease Control and Prevention (CDC) 2008 revised surveillance case definitions: A documented AIDS diagnosis, report of any AIDS-defining condition within 24 months prior to or six months following a diagnosis of HIV, or a documented CD4+ T-lymphocyte count of <200cells/μL qualified a patient as having AIDS.^10^ Those patients who did not have documentation of a CD4 count within six months of their diagnosis of HIV were classified as non-AIDS status.

### Statistical Analysis

We compared demographic characteristics of patients tested in the two testing site types (outpatient versus inpatient) using chi-square tests to determine whether the sites served the same populations (Table 1). We then conducted logistic regression to assess the relationship between socioeconomic, demographic and biomedical factors and linkage to care. Socioeconomic and demographic factors assessed included gender, race, age, employment, housing and insurance. Biomedical factors included initial CD4 and viral load collected within six months of new diagnosis, current substance use, and diagnosis of mental illness.

We used simple logistic regression to test for associations between any single variable and the primary outcome variable, failure to link to care (Table 2). Those variables found to be significant at the univariate level (p<0.05) were subsequently included in the multivariate models. In the first logistic regression analysis, we entered each of the significant variables from the univariate models singly into a two-predictor model and controlled for testing site. We employed an additional multivariate model that included all variables associated with failure to link to care in univariate analyses, including testing site (Table 3). Statistical testing was two-sided, and we considered (p<0.05) statistically significant. All models were tested using Pearson’s goodness-of-fit tests, and considered satisfactory if the p-value for the test statistic was greater than 0.05.^11^ Model results are presented for prediction of failure to link to care, rather than successful linkage, because it is the failure to link that is critical to identify. We omitted patients with missing data from the analyses. Analyses were performed using Stata IC Version 12.

## RESULTS

Between May 2007 and November 2011, 5,886 tests were conducted in the hospital-based setting (ED and inpatient units); 682 tests were done in the DUCOM outpatient primary care clinics. Of the combined 6,568 patients, 96 were newly diagnosed with HIV, with an overall seropositivity rate of 1.46%. Of the 96 positives, 87 (63 inpatient and 24 outpatient) were eligible for the study. We excluded nine for the following reasons: seven had inadequate information in the medical records; one died prior to hospital discharge, one was discharged to hospice care (Figure).

Overall, 63 (72%) patients successfully linked to care following a new HIV diagnosis and 24 (28%) did not link to care. Of patients diagnosed in the outpatient setting 23 (96%) linked to care, and 40 (63%) from the hospital-based setting (ED or inpatient) linked to care (p<0.01).

Participants were largely male (67%), black (87%), and age 30 or older (68%). Fifty-three (61%) were unemployed, 21 (24%) were homeless, 26 participants (30%) were using drugs or alcohol, 17 (20%) had a history of mental illness, and 28 (32%) were uninsured (Table 1). Those who were tested in the hospital-based settings were more likely to be black (p=0.01), homeless (p=0.03), and use alcohol or drugs (p=0.03) than those tested in the outpatient clinics.

At the time of testing, 31 participants (36%) were diagnosed with AIDS. Of these 31 patients, 19 had clinical symptoms. There were nine cases of pneumocystis jirovecii pneumonia, three cases of HIV wasting syndrome, two cases of esophageal candidiasis, and one case of each of the following: cryptosporidiosis, cryptococcal meningoencephalitis, toxoplasmosis of the central nervous system, Kaposi’s sarcoma, and Burkitt’s lymphoma. Only three of the symptomatic patients had CD4+ T-lymphocyte counts above 200cells/μL, and all patients with AIDS had a CD4+ count under 300cells/μL. The remaining 12 patients with AIDS were asymptomatic, but had CD4+ T-lymphocyte counts below 200cells/μL. Eighteen patients did not have a documented CD4 count or known AIDS-defining illness, and thus were combined with the HIV+ but non-AIDS patients.

On a univariate level, unemployment (OR: 11.4, 95% CI [2.46–54.41], p<0.01), homelessness (OR: 3.38 95%, CI [1.19–9.55], p=0.02), current substance use (OR: 7.88, 95% CI [2.75–22.55], p<0.01), mental illness (OR: 3.20, 95% CI [1.05–9.72], p=0.045), and testing site (hospital based verses outpatient clinic) (OR: 13.2, 95% CI [1.67–104.47], p<0.01) were all associated with failure to link to care (Table 2).

Results from the full multivariate model indicated that patients diagnosed with HIV in the hospital ED and inpatient units had higher odds of failure to link to care (OR: 10.9, 95% CI [1.26–94.01], p=0.03) than patients diagnosed in the outpatient clinics. Additionally, unemployment was significantly associated with failure to link to care (OR: 6.50, 95% CI [1.13–37.32], p=0.04). When testing site was controlled in the two-predictor model, however, the odds of failure to link to care were significantly increased by unemployment (OR: 12.2, 95% CI [2.54–58.16], p<0.01) and current substance use (OR: 6.44, 95% CI [2.15–19.30], p<0.01) (Table 3).

Of the eligible patients, 59 (67.8%) met with a representative from the HIV clinic and received an appointment for follow up at the time of their diagnoses, and 28 (32%%) were given a follow-up appointment without meeting a representative from the HIV clinic. Those who met with an HIV representative at diagnosis were equally likely to fail to link to care (27.1% vs 28.6%, p=0.89). The ED patients linked to care at a similar rate (65%) as those who were inpatients (56%) (p=0.56).

## DISCUSSION

In the present study, receiving a new HIV diagnosis in a hospital-based setting (inpatient unit or ED) as compared to an outpatient medical setting, was associated with failure to link to care. Overall, 72% of patients successfully linked to care following a new HIV diagnosis, while 28% did not link, which is similar to previous findings.^4^ Nearly all patients (96%) from the outpatient setting linked to care, compared to 63% of patients from the hospital-based setting. Our findings are in contrast to those of a large meta-analysis, in which EDs and urgent care centers had higher linkage rates than community-based settings.^11^ The community-based testing sites, however, were rather heterogenous, which makes a direct comparison between our findings difficult.

Our two populations differed in that patients who tested in the hospital-based setting were more likely than those who were tested in the out-patient clinic to be black, homeless, and use alcohol or drugs. Of these individual variables however, only being homeless was found to be significant in predicting linkage to care at the multivariate level. When testing site was controlled, unemployment and substance use were found to be associated with failure to link, which is consistent with previous work.^12^–^15^ While female gender, racial/ethnic minority, lack of insurance, and mental illness have been identified as predictors of failure to establish HIV care in other studies^16^–^18^ our evaluation did not show such a relationship. Other potential barriers, such as poverty, fear of death, stigma, and violence from a domestic partner,^16^ were not specifically explored in our study, but such issues could have impacted why those who tested in the hospital were less likely to link to care.

Our results indicate that a health disparity may exist between those who access care in the ED and those who access care in the outpatient setting. Patients tested in the outpatient setting were presumably familiar with Drexel staff, accessing outpatient clinics, and following administrative procedures. It may be that this familiarity with the outpatient setting is an important factor in linkage to care, and may have fostered such success in linkage to HIV care compared to those tested in the hospital where there was no established relationship. Unfortunately, neither of the interactions that involved meeting of case managers or HIV consult service with newly-diagnosed patients from the ED or inpatient units appeared to impact rates of linkage to HIV care, suggesting that such “face-time” between patients and clinic staff was not sufficient to engage new patients. Our results demonstrate the acceptability and benefit of opt-out testing in an outpatient setting with 96% successful linkage to care. Additionally, the rate of linkage to care from the ED (65%) indicates that this setting has the potential to have successful linkages with enhanced interventions in place.

A sizable proportion of persons with HIV in the U.S. are not achieving successful HIV treatment due to failures at early steps along the HIV treatment cascade.^4^ While entry into care after an HIV diagnosis, defined as a visit with an HIV care provider authorized to prescribe ART, has been associated with improved survival,^19^ only 69% of those who know that they are infected with HIV are linked to care.^20^ Efforts to link patients must address structural barriers identified in this study such as homelessness, unemployment, substance use, and mental illness. The International Association of Physicians in AIDS Care has developed guidelines for improving entry into and retention in care for persons with HIV. These guidelines recommend brief strengths-based case management interventions, intensive outreach for individuals not engaged in care within six months of a new HIV diagnosis, and use of peer patient navigators as a model of care coordination as ways to improve linkage and retention into HIV care.^21^ In our study, we utilized case management and outreach personal to engage those with a new HIV diagnosis and those who missed appointments within six months of the new HIV diagnosis. While linkage rates were positive in this study, using strengths-based case management interventions and peer patient navigators may have further improved these rates. Strength-based case management encourages patient participation in setting treatment goals and works to resolve patient-identified barriers to treatment. [Substance Abuse and Mental Health Services Administration National Registry of Evidenced based programs and practices: Brief Strengths-Based Case Management http://www.nrepp.samhsa.gov/ViewIntervention.aspx accessed April 21, 2015.]

In this study, a high percentage of persons with a new HIV diagnosis had a diagnosis of AIDS (36%). The likelihood of having AIDS did not differ between the inpatient or outpatient setting, and having an AIDS diagnosis did not predict linkage to care. These results are consistent with U.S. numbers in that 32% of persons found to have HIV in 2008 received a diagnosis of AIDS within 12 months of their initial HIV diagnosis.^22^ Late presentation for HIV medical care results in considerable morbidity and mortality with a 9–14 fold increased one-year mortality among patients with initial CD4 counts less than 200 cells/μl.^23^ Our study demonstrates the importance of testing in medical settings in order to achieve more timely testing and linkage to care.^24^

## LIMITATIONS

There are several limitations to this study that may limit the generalizability of the results. Because of the small sample size and low sero-positivity rate (1.46%), the actual number of individuals detected and included in the study is low. The final results may have differed if we were able to include the seven excluded patients who had inadequate information in the medical records. We were also unable to determine if those patients who did not keep appointments at the co-located HIV clinic attained care at another HIV clinic or with a primary care provider, perhaps underestimating linkage rates. Additionally, there were differences in patient demographics between the outpatient and hospital-based testing groups, in particular there were higher rates of homelessness in the ED. This may have impacted the primary outcome of linkage to care between the two settings. It would be useful to know if those patients who were diagnosed at the hospital-based site had a primary care provider, so that barriers to testing could be further explored (ie: Why were they not tested in their clinics?). The number of participants from the outpatient clinics was smaller than the hospital-based participants. It would be beneficial for future studies to examine the linkage rate of the differing populations between the ED and the inpatient unit in order to explore interventions that may be unique to each of these setting. Additionally, future studies would benefit from a larger sample size to further clarify the role of the ED as an entry point to care.

Although the protocol for screening was an opt-out model, there may have been differences in the screening process at the different sites. For example, the low numbers of tests employed in the outpatient clinics bring into question the true employment of “opt-out” testing at these sites.

## CONCLUSION

As the United States struggles with the challenges of successful linkage to care of newly diagnosed HIV individuals, models of testing in outpatient medical clinics need to be considered. The comparative success of 96% successful linkage to care in the outpatient medical setting as demonstrated in this study highlights the opportunity and benefit of routine opt-out testing in primary care practices. The coordination of personnel in the ED with co-located HIV clinics to facilitate linkage to care is recommended. Further research is needed to better understand perceived barriers that prevent effective linkage and engagement in care for this largely vulnerable population. To decrease the rate of HIV transmission in the U.S., increased HIV testing and linkage to care must be strengthened. The results from this study will inform and provide direction for future research in HIV linkage to care. To fully maximize the benefits of expanded HIV testing will require careful implementation, adaptation, and evaluation of linkage-to-care programs.

## Figures and Tables

**Figure:**
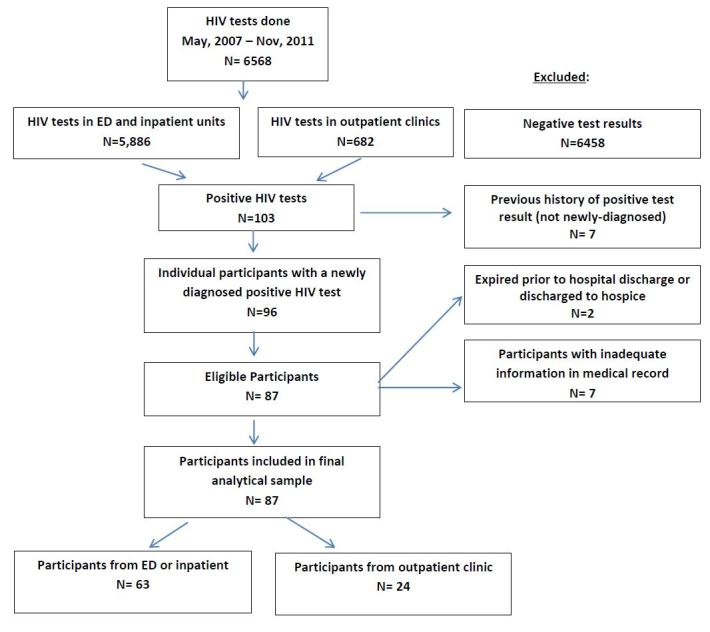
Subject disposition. *HIV*, human immunodeficiency virus; *ED,* emergency department

**Table 1:** Demographic characteristics of HIV-positive patients tested in outpatient versus inpatient settings.

Variable	Total n (%), n=87	Outpatient (%), n=24	Inpatient and ED (%), n=63	Pearson chi-square test p-value
Sex				
Female	29 (33%)	9 (38%)	20 (32%)	0.61
Male	58 (67%)	15 (63%)	43 (68%)	0.61
Race				
Black	76 (87%)	17 (71%)	59 (94%)	0.01
White	7 (8%)	4 (17%)	3 (5%)	0.01
Other	4 (5%)	3 (13%)	1 (2%)	0.01
Age				
18–29	28 (32%)	11 (46%)	17 (27%)	0.09
30–65	59 (68%)	13 (54%)	46 (73%)	0.09
Unemployed	53 (61%)	13 (54%)	40 (63%)	0.43
Homeless	21 (24%)	2 (8%)	19 (30%)	0.03
Substance abuse	26 (30%)	3 (13%)	23 (37%)	0.03
Mental illness	17 (20%)	2 (8%)	15 (24%)	0.10
Uninsured	28 (32%)	4 (17%)	24 (38%)	0.06
AIDS	31 (36%)	6 (25%)	25 (40%)	0.06

*HIV,* human immunodeficiency virus; *ED,* emergency department; *AIDS*, acquired immune deficiency syndrome

**Table 2:** Linkage to care in single logistic regression.

Variable	Linked to care (%) n=63*	Did not link to care (%) n=24*	Pearson chi-square test p-value	OR (95% CI)
Sex				
Female	19 (66%)	10 (34%)	0.31	1.65 (0.62,4.38)
Male	44 (76%)	14 (24%)	0.31	reference
Race				
White	6 (86%)	1 (14%)	0.30	reference
Black	53 (70%)	23 (30%)	0.30	2.60 (0.30,22.87)
Other	4 (100%)	0 (0%)	0.30	N/A
Age				
>30 years	39 (68%)	18 (32%)	0.25	1.85 (0.64,5.30)
≤30 years	24 (80%)	6 (20%)	0.25	reference
Unemployed				
Yes	31 (58%)	22 (42%)	<0.01	11.4 (2.46,54.41)
No	32 (94%)	2 (6%)	<0.01	reference
Uninsured				
Yes	19 (68%)	9 (32%)	0.51	1.39 (0.52,3.72)
No	44 (75%)	15 (25%)	0.51	reference
Homeless				
Yes	11 (52%)	10 (48%)	0.02	3.38 (1.19,9.55)
No	52 (79%)	14 (21%)	0.02	reference
Substance abuse				
Yes	11 (42%)	15 (58%)	<0.01	7.88 (2.75,22.55)
No	52 (85%)	9 (15%)	<0.01	reference
Mental illness				
Yes	9 (53%)	8 (47%)	0.045	3.20 (1.05,9.72)
No	54 (78%)	16 (23%)	0.045	reference
AIDS				
Yes	26 (84%)	5 (16%)	0.76	1.23 (0.32,4.72)
No	32 (86%)	5 (14%)	0.76	reference
Testing site				
Outpatient	23 (96%)	1 (4%)	<0.01	reference
Hospital (ED or inpatient)	40 (63%)	23 (37%)	<0.01	13.2 (1.67, 104.47)

*ED,* emergency department; *AIDS*, acquired immune deficiency syndrome

* 72% linked to care, 28% did not link to care.

**Table 3:** Correlates of unsuccessful linkage to care in multivariate models.*

	Multivariate (controlled for ED/inpatient testing site)			Multivariate (full model)		
		
Variable	OR	95% CI	p-value	OR	95% CI	p-value
Unemployment	12.2	2.54, 58.16	<0.01	6.50	1.13, 37.32	0.04
Homelessness	2.50	0.85, 7.40	0.10	1.18	0.34, 4.07	0.80
Substance use	6.44	2.15, 19.30	<0.01	2.72	0.75, 9.90	0.13
Mental illness	2.36	0.74, 7.50	0.14	1.06	0.28, 4.02	0.93
Testing site (not outpatient)	-	-	-	10.9	1.26, 94.01	0.03

*ED,* emergency department

* All variables used had p<0.05 in the single logistic regression.
